# Supplementary material for: Parallel development of social behavior in biological and artificial fish
Source: Nat Commun. 2024 Dec 5;15:10613. doi: 10.1038/s41467-024-52307-4 (PMC11621320; doi:10.1038/s41467-024-52307-4)
Supplement: Supplementary file 1 — Supplementary Information [file 41467_2024_52307_MOESM1_ESM.pdf]

Supplementary Information:
Parallel development of social behavior in biological and artificial fish

Joshua D. McGraw*, Donsuk Lee, & Justin N. Wood*

*To whom correspondence should be addressed: jdmcgraw@iu.edu or woodjn@iu.edu

This PDF file includes:

Figs. S1-S5

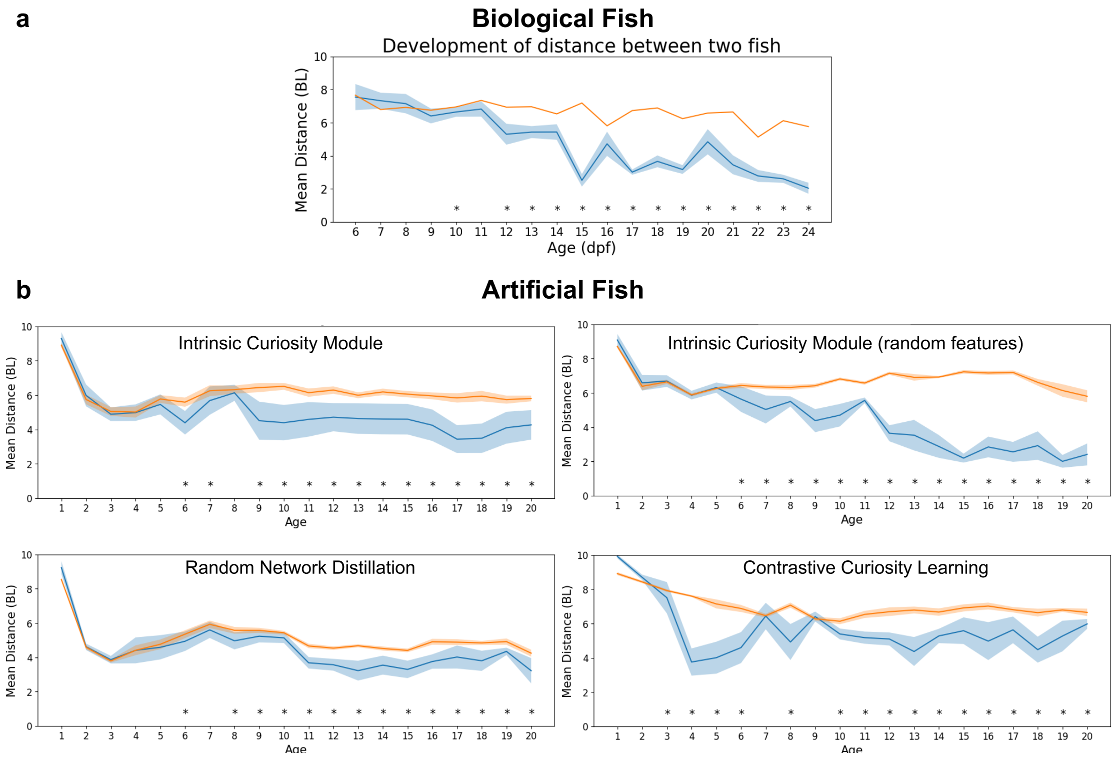

Figure S1: Development of distance and relative position between two fish. (a) Most likely distance between pairs of biological fish (blue lines) at ages 6 dpf to 24 dpf, along with control randomized data (orange lines). (b) Most likely distance between pairs of artificial fish (blue lines) along with control randomized data (orange lines). For each intrinsic reward algorithm, we trained 20 artificial fish together in a virtual fish tank and saved the weights in their neural networks at 20 equally spaced time points during training. "Age" is defined as the index of the checkpoint in chronological order. Two consecutive checkpoints were 40,000 time steps apart. For testing, we grouped the fish into 10 pairs and placed each pair in the virtual test tank. Data are presented as mean values. Error bars indicate ± 1 SEM. Data from the biological fish (Panel A) reproduced from Hinz & de Polavieja[43].

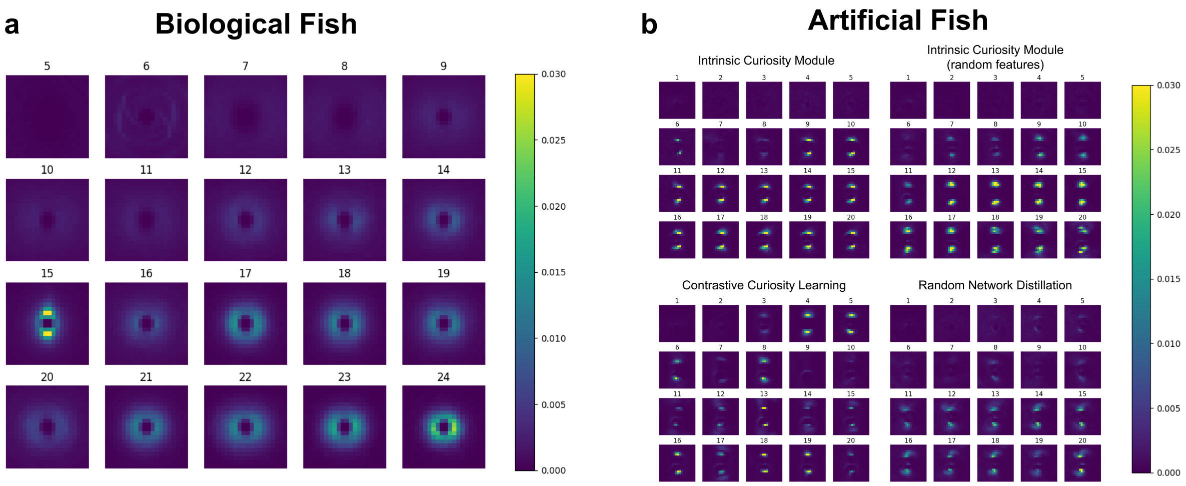

Figure S2: Development of relative position between two fish. (a) Probability of finding neighbors at different relative positions for biological fish, shown as a function of age (6 dpf to 24 dpf). The focal animal is at the center of the coordinate system, with the velocity vector pointing in the direction of the y axis. (b) Probability of finding neighbors at different relative positions for artificial fish, shown as a function of training "age". For both biological and artificial fish, the probability of finding a nearby neighbor increases across the learning (training) phase. Data from the biological fish (Panel A) reproduced from Hinz & de Polavieja[43].

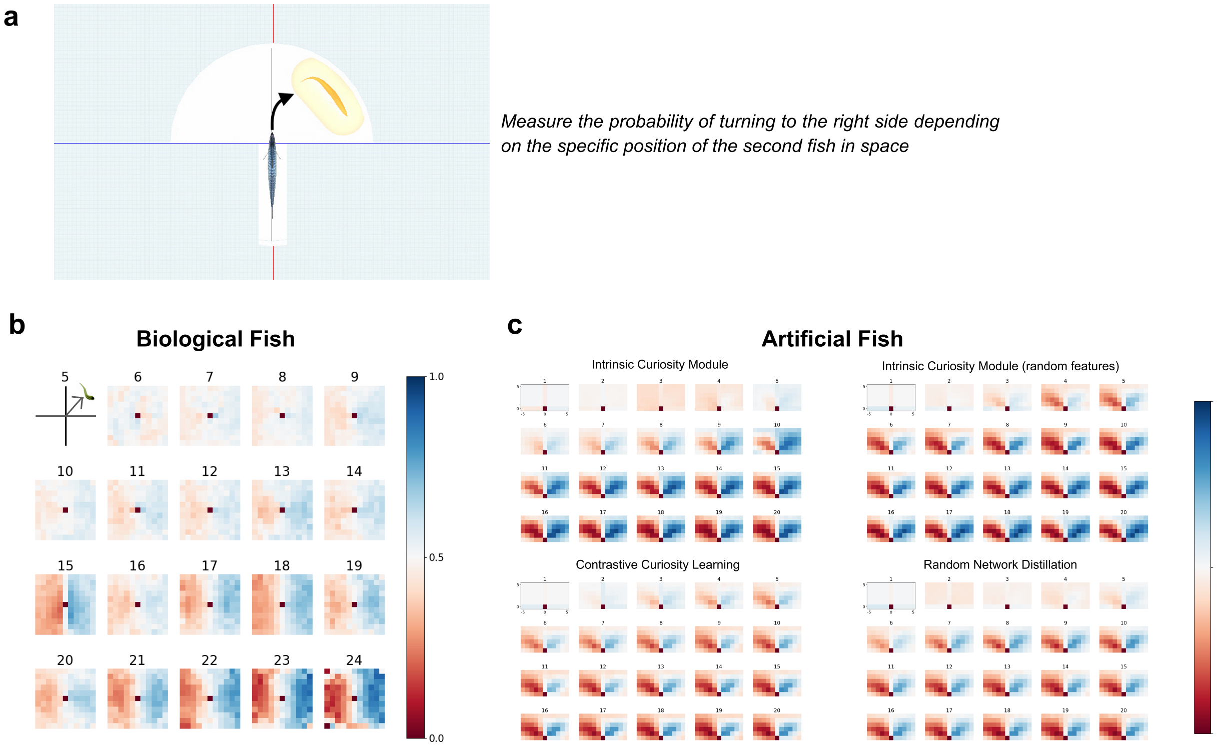

Figure S3: (a) We measured social attraction by studying the probability of turning to the right side depending on the position of the second fish in space. The (b) biological fish and (c) artificial fish developed common sensitivities to the relative position of other fish in space. Data are presented as mean values. Data from the biological fish (Panel B) reproduced from Hinz & de Polavieja[43].

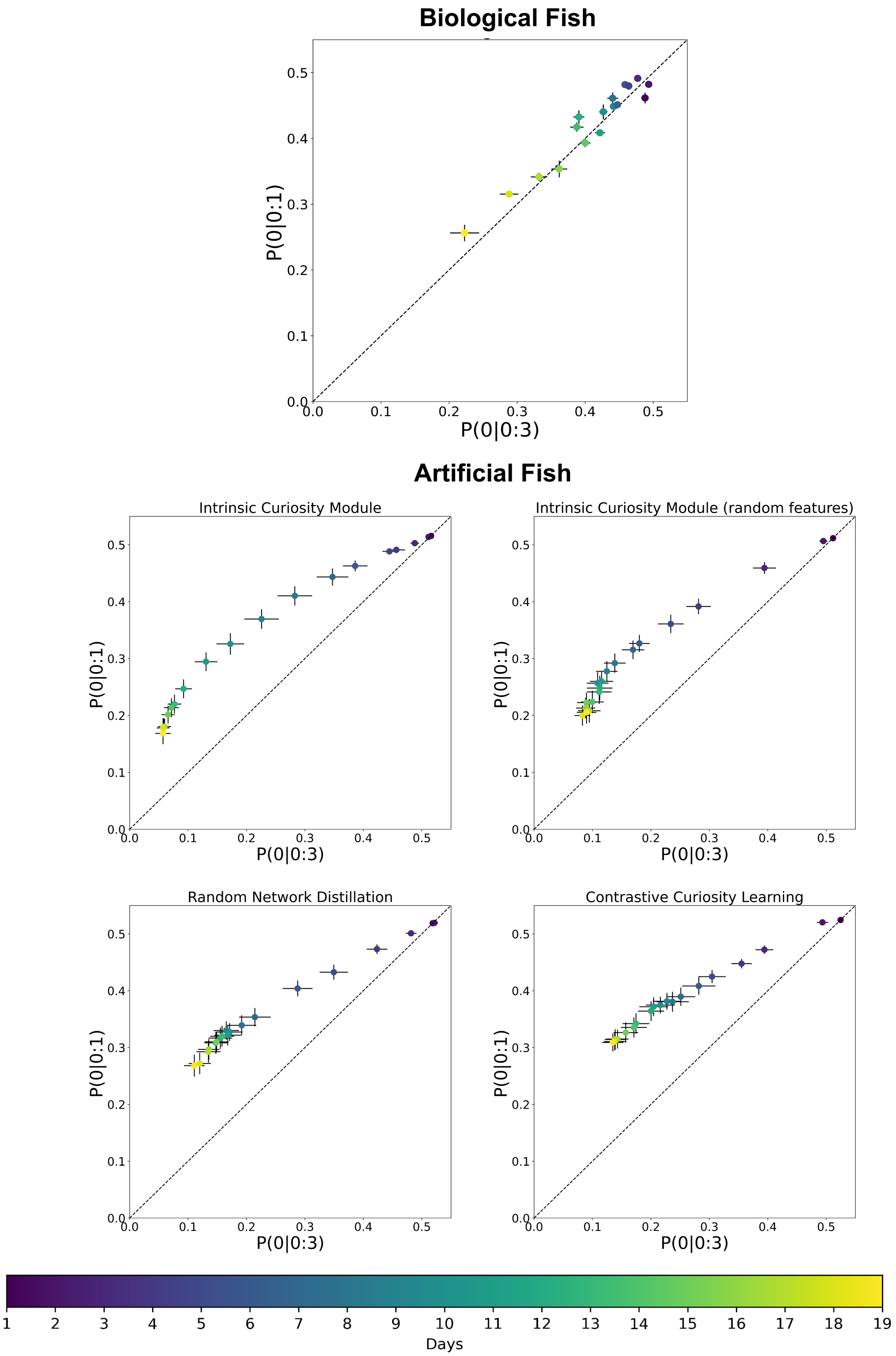

Figure S4: Relationship between the probability of turning to the side with no animals in experiments with two and four fish, P(0 | 0:1) vs. P(0 | 0:3) for the biological fish (top) and artificial fish (bottom: 4 intrinsic motivation algorithms). Dots indicate experimental data (color coded by age), and dashed lines indicate the theoretical prediction derived from the interaction rule in Hinz & de Polavieja[43]. Data from the biological fish reproduced from Hinz & de Polavieja[43].

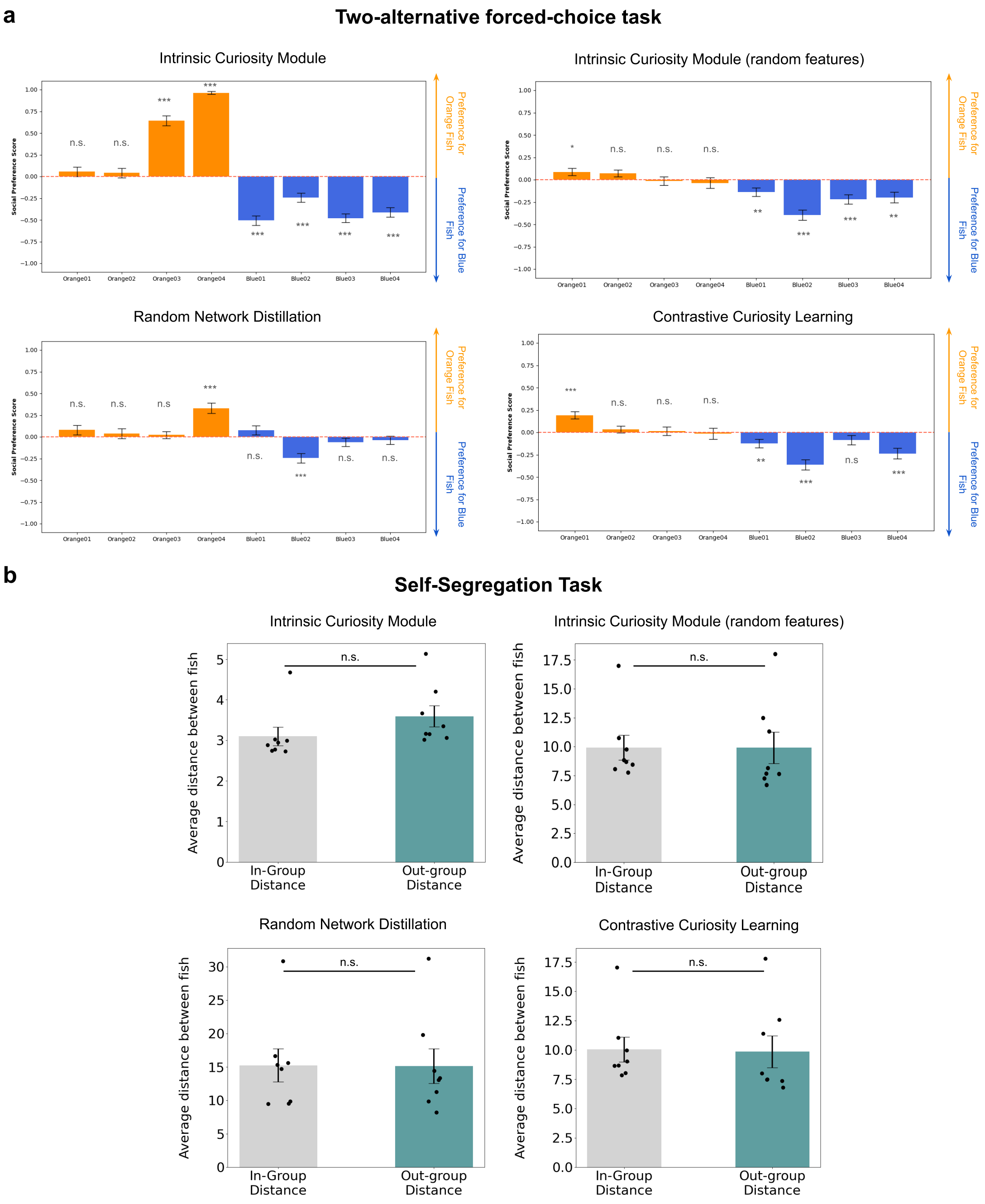

Figure S5: Reducing intrinsic motivation. (a) Compared to the artificial fish with high levels of intrinsic motivation (data shown in Fig. 6b), the artificial fish with low intrinsic motivation showed little to no evidence of social grouping in the 2AFC task. One-sample t-tests were performed to determine statistical significance. (b) Likewise, contrary to the artificial fish with high levels of intrinsic motivation (data shown in Fig. 7b), the artificial fish with low intrinsic motivation spent equal amounts of time with in-group versus out-group members in the self-segregation task. The development of social preferences in the artificial fish required high intrinsic motivation. Paired samples t-tests (two-tailed) were performed to determine statistical significance. For both panels: $*p \leq 0.05$, $**p \leq 0.01$, and $***p \leq 0.001$, uncorrected; the exact p values and raw values are provided in the Source Data. Black dots indicate performance of individual artificial fish. Data are presented as mean values. Error bars indicate ± 1 SEM.
