# Supplementary material for: Parallel development of social behavior in biological and artificial fish
Source: Nat Commun. 2024 Dec 5;15:10613. doi: 10.1038/s41467-024-52307-4 (PMC11621320; doi:10.1038/s41467-024-52307-4)
Supplement: Supplementary file 3 — Description of Additional Supplementary Files [file 41467_2024_52307_MOESM3_ESM.pdf]

File name: Supplementary Movie 1

Description: Grouping behavior of the artificial fish across the training phase. The artificial fish shown here had the intrinsic curiosity learning algorithm. Training 'day' refers to evenly-spaced checkpoints during the training phase, which mimics the 24 rearing days in the biological fish study. Early in training, the artificial fish did not show evidence of grouping behavior, akin to real fish. During middle and late stages of training, the artificial fish (like real fish) spontaneously learned grouping behavior and followed one another around the virtual fish tank.
