# Supplementary material for: Parallel development of social behavior in biological and artificial fish
Source: Nat Commun. 2024 Dec 5;15:10613. doi: 10.1038/s41467-024-52307-4 (PMC11621320; doi:10.1038/s41467-024-52307-4)
Supplement: Supplementary file 5 — Reporting Summary [file 41467_2024_52307_MOESM5_ESM.pdf]

Corresponding author(s): Joshua D. McGraw
Justin N. Wood

Last updated by author(s): Jul 16, 2024

Reporting Summary

Nature Portfolio wishes to improve the reproducibility of the work that we publish. This form provides structure for consistency and transparency in reporting. For further information on Nature Portfolio policies, see our Editorial Policies and the Editorial Policy Checklist.

Statistics

For all statistical analyses, confirm that the following items are present in the figure legend, table legend, main text, or Methods section.

n/a	Confirmed	
☐	☒	The exact sample size (*n*) for each experimental group/condition, given as a discrete number and unit of measurement
☐	☒	A statement on whether measurements were taken from distinct samples or whether the same sample was measured repeatedly
☐	☒	The statistical test(s) used AND whether they are one- or two-sided<br>*Only common tests should be described solely by name; describe more complex techniques in the Methods section.*
☐	☒	A description of all covariates tested
☐	☒	A description of any assumptions or corrections, such as tests of normality and adjustment for multiple comparisons
☐	☒	A full description of the statistical parameters including central tendency (e.g. means) or other basic estimates (e.g. regression coefficient) AND variation (e.g. standard deviation) or associated estimates of uncertainty (e.g. confidence intervals)
☐	☒	For null hypothesis testing, the test statistic (e.g. *F*, *t*, *r*) with confidence intervals, effect sizes, degrees of freedom and *P* value noted<br>*Give P values as exact values whenever suitable.*
☒	☐	For Bayesian analysis, information on the choice of priors and Markov chain Monte Carlo settings
☒	☐	For hierarchical and complex designs, identification of the appropriate level for tests and full reporting of outcomes
☒	☐	Estimates of effect sizes (e.g. Cohen's *d*, Pearson's *r*), indicating how they were calculated

Our web collection on statistics for biologists contains articles on many of the points above.

Software and code

Policy information about availability of computer code

Data collection	The virtual experiments were run in the Unity Game Engine. We used a package known as ML-Agents Toolkit. We used the following software: Unity ML-Agents version 2.0.1, Python 3.8.10 with PyTorch 1.7.1+cu110, Python 'mlagents' library version 0.26.0, 'ml-agents-envs' version 0.26.0, and ML-agents' Communicator API 1.5.0.
Data analysis	The data and code used for training and testing artificial fish is available on our GitHub page: https://github.com/buildingamind/McGraw_Lee_Wood_2024

For manuscripts utilizing custom algorithms or software that are central to the research but not yet described in published literature, software must be made available to editors and reviewers. We strongly encourage code deposition in a community repository (e.g. GitHub). See the Nature Portfolio guidelines for submitting code & software for further information.

Data

Policy information about availability of data

All manuscripts must include a data availability statement. This statement should provide the following information, where applicable:

- Accession codes, unique identifiers, or web links for publicly available datasets
- A description of any restrictions on data availability
- For clinical datasets or third party data, please ensure that the statement adheres to our policy

All of the source data used to create the figures in this paper are available as a Source Data file. Source data are provided with this paper.

Research involving human participants, their data, or biological material

Policy information about studies with human participants or human data. See also policy information about sex, gender (identity/presentation), and sexual orientation and race, ethnicity and racism.

Reporting on sex and gender	N/A: We had no original human or animal data in our study.
Reporting on race, ethnicity, or other socially relevant groupings	N/A: We had no original human or animal data in our study.
Population characteristics	N/A: We had no original human or animal data in our study.
Recruitment	N/A: We had no original human or animal data in our study.
Ethics oversight	N/A: We had no original human or animal data in our study.

Note that full information on the approval of the study protocol must also be provided in the manuscript.

Field-specific reporting

Please select the one below that is the best fit for your research. If you are not sure, read the appropriate sections before making your selection.

☐ Life sciences ☒ Behavioural & social sciences ☐ Ecological, evolutionary & environmental sciences

For a reference copy of the document with all sections, see nature.com/documents/nr-reporting-summary-flat.pdf

Behavioural & social sciences study design

All studies must disclose on these points even when the disclosure is negative.

Study description	We compared the development of grouping behavior and social preferences across biological and artificial fish. The data were quantitative. The data for the biological fish were collected in prior studies, as indicated in the manuscript.
Research sample	The research subjects were embodied deep neural networks (artificial fish). We compared the behavior of the artificial fish to the data from biological fish collected in prior studies.
Sampling strategy	The artificial fish were randomly assigned to the experimental conditions. The brains (ANNs) in the artificial fish were randomly initialized at the start of the training phase. Our sample sizes for the artificial fish were based on the target animal studies (previously published studies exploring the development of collective behavior and social preferences in biological fish). We used the same sample sizes as those target animal studies.
Data collection	All data collection was performed in a video game engine (Unity 3D). The experimenters had no control over the behavior of the artificial fish. All data collection procedures were automated in the video game engine.
Timing	We started collecting data around Jan. 2021 and stopped collecting data in Jan. 2024.
Data exclusions	No data were excluded from the manuscript.
Non-participation	No subjects were excluded from the manuscript.
Randomization	The artificial fish were randomly assigned to the experimental groups.

Reporting for specific materials, systems and methods

We require information from authors about some types of materials, experimental systems and methods used in many studies. Here, indicate whether each material, system or method listed is relevant to your study. If you are not sure if a list item applies to your research, read the appropriate section before selecting a response.

Materials & experimental systems

n/a	Involved in the study
☒	☐ Antibodies
☒	☐ Eukaryotic cell lines
☒	☐ Palaeontology and archaeology
☒	☐ Animals and other organisms
☒	☐ Clinical data
☒	☐ Dual use research of concern
☒	☐ Plants

Methods

n/a	Involved in the study
☒	☐ ChIP-seq
☒	☐ Flow cytometry
☒	☐ MRI-based neuroimaging

Plants

Seed stocks

Report on the source of all seed stocks or other plant material used. If applicable, state the seed stock centre and catalogue number. If plant specimens were collected from the field, describe the collection location, date and sampling procedures.

Novel plant genotypes

Describe the methods by which all novel plant genotypes were produced. This includes those generated by transgenic approaches, gene editing, chemical/radiation-based mutagenesis and hybridization. For transgenic lines, describe the transformation method, the number of independent lines analyzed and the generation upon which experiments were performed. For gene-edited lines, describe the editor used, the endogenous sequence targeted for editing, the targeting guide RNA sequence (if applicable) and how the editor was applied.

Authentication

Describe any authentication procedures for each seed stock used or novel genotype generated. Describe any experiments used to assess the effect of a mutation and, where applicable, how potential secondary effects (e.g. second site T-DNA insertions, mosiacism, off-target gene editing) were examined.
